# Rates and Associated Factors of Secondary Mental Health Care Utilisation among Ex-Military Personnel in the United States: A Narrative Review

**DOI:** 10.3390/healthcare7010018

**Published:** 2019-01-28

**Authors:** Katharine M. Mark, Dominic Murphy, Sharon A.M. Stevelink, Nicola T. Fear

**Affiliations:** 1 King’s Centre for Military Health Research, King’s College London, Weston Education Centre, Cutcombe Road, London SE5 9RJ, UK; katharine.mark@kcl.ac.uk (K.M.M.); dominic.murphy@combatstress.org.uk (D.M.); nicola.t.fear@kcl.ac.uk (N.T.F.); 2 Combat Stress, Tyrwhitt House, Oaklawn Road, Leatherhead, Surrey KT22 0BX, UK; 3 Department of Psychological Medicine, King’s College London, Institute of Psychiatry, Psychology and Neuroscience, De Crespigny Park, London SE5 8AF, UK; 4 Academic Department of Military Mental Health, King’s College London, Weston Education Centre, Cutcombe Road, London SE5 9RJ, UK

**Keywords:** mental health care utilisation, mental health, secondary mental health care, post-traumatic stress disorder, veterans, narrative review, help-seeking

## Abstract

Little is known about ex-serving military personnel who access secondary mental health care. This narrative review focuses on studies that quantitatively measure secondary mental health care utilisation in ex-serving personnel from the United States. The review aimed to identify rates of mental health care utilisation, as well as the factors associated with it. The electronic bibliographic databases OVID Medline, PsycInfo, PsycArticles, and Embase were searched for studies published between January 2001 and September 2018. Papers were retained if they included ex-serving personnel, where the majority of the sample had deployed to the recent conflicts in Iraq or Afghanistan. Fifteen studies were included. Modest rates of secondary mental health care utilisation were found in former military members—for mean percentage prevalence rates, values ranged from 12.5% for at least one psychiatric inpatient episode, to 63.2% for at least one outpatient mental health appointment. Individuals engaged in outpatient care visits most often, most likely because these appointments are the most commonly offered source of support. Post-traumatic stress disorder, particularly re-experiencing symptoms, and comorbid mental health problems were most consistently associated with higher mental health care utilisation. Easily accessible interventions aimed at facilitating higher rates of help seeking in ex-serving personnel are recommended.

## 1. Introduction

### 1.1. Background

The estimated size of the United States’ (U.S.) ex-serving military population is approximately 20 million [1]. A number of former service personnel may experience mental health problems [2]. Indeed, in a large population-based study of over 300,000 Army soldiers and Marines who completed the post-deployment health assessment six months after returning from active duty, 13% of personnel were experiencing major depression, post-traumatic stress disorder (PTSD), suicidal or aggressive ideation, or interpersonal conflict [3]. The U.S. Department of Veterans Affairs (VA) has shown that mental health problems in U.S. ex-serving personnel may have increased over time, with up to 26% of veterans reporting difficulties [4].

Previous research suggests that between 23% and 40% of former military members who meet the criteria for a psychiatric difficulty access mental health services when needed [5]. A wide range of factors influence this group’s treatment-seeking, including a lack of recognition of their mental health disorder symptoms; believing that their symptoms are not severe enough; a propensity to favour informal over formal sources of help; preferring to deal with problems oneself; fearing adverse occupational outcomes; previous negative treatment experiences; problems accessing services; and concern about the stigma associated with mental ill health [6,7,8,9,10,11].

Of the ex-serving personnel who do seek treatment, around 20% receive medication or counselling [5,7], typically by entering the health care system at primary care level—defined as physician or nurse practitioners, or community-based services. One previous study found that most U.S. ex-service members who present to care in this context tend to be unemployed, with negative beliefs about mental health, and poor social support and daily functioning [12]. While it is important to know the profile of those who take the first step to tackling their problem(s) through primary care, there is a lack of evidence concerning former military personnel who access secondary mental health care services—defined as more specialist care, frequently delivered in therapeutic clinics, or hospitals. Those who access secondary mental health services, or who are referred to these services from primary care, will, in general, have more complex levels of need compared to those who remain in primary care. We currently know little about the numbers of ex-service men and women utilising secondary mental health care, and the factors associated with such utilisation.

### 1.2. Objectives

While there have been reviews of treatment-seeking for mental health problems experienced by currently serving U.S. military personnel [6], there have, to the best of our knowledge, been none focusing solely on ex-serving military personnel accessing secondary mental health care treatment. To address this gap, we narratively reviewed studies, published between 2001 and 2018, that quantitatively measure secondary mental health care utilisation in U.S. ex-serving men and women. The review aimed to identify rates of mental health care utilisation, as well as the factors associated with such utilisation. We employed a narrative review design, as opposed to a systematic review design, in order to provide a broad and descriptive overview of this research area.

## 2. Materials and Methods

### 2.1. Search Strategy

We searched a range of electronic databases, including OVID Medline (Ovid Technologies, Inc., New York, NY, U.S.), PsycInfo (American Psychological Association, Washington, DC, U.S.), PsycArticles (American Psychological Association, Washington, DC, U.S.), and Embase (Elsevier, Amsterdam, The Netherlands), to identify papers published in English that reported on secondary mental health care utilisation among ex-military personnel. A combination of the following key words were used: veteran, military, active duty, soldier, service member, ex-serving, ex-military, armed forces, mental health, mental well-being, mental disorder, treatment-seeking, treatment use, help-seeking, and service use. Searches were limited to articles published between January 2001 and September 2018—to allow a focus on recent mental health care utilisation over a 16-year period, and to cover the entirety of the conflicts in Iraq and Afghanistan (and any papers published subsequently). The reference lists of eligible articles were checked for any further papers that fitted the study criteria.

### 2.2. Eligibility Criteria

The inclusion criteria for the review were: (1) studies quantitatively measuring mental health care utilisation as their outcome; (2) studies focusing on secondary mental health care—defined as specialist care, usually delivered in therapeutic clinics, or hospitals, and including VA medical facilities; and (3) studies focusing on ex-military samples only, where the majority of included personnel had been deployed to the conflict(s) in Iraq and/or Afghanistan.

The exclusion criteria for the review were: (1) reviews, case studies, conference proceedings, books, theses, dissertations, and comments on/corrections to/replies to original papers; (2) studies (including randomised controlled trials) testing/investigating the use of specific clinical interventions; (3) studies focusing on informal mental health care utilisation—defined as support received through a partner, family, friends, colleagues, or helplines; and (4) studies focusing on specific subgroups of the (ex-) military—in particular, those related to: sexual trauma or violence, homelessness, the criminal justice system, and disability claims or compensation.

## 3. Results

**Figure 1:**
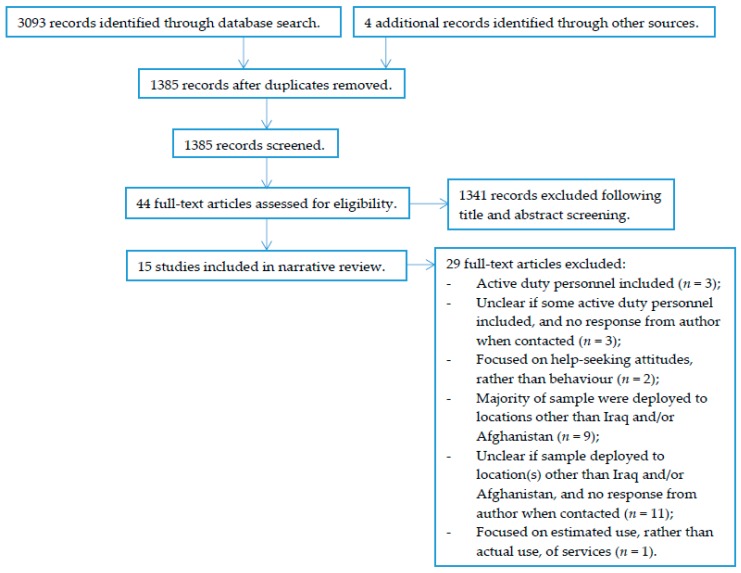
The article selection strategy used.

A total of 15 papers were retained (see Figure 1). While we searched for papers conducted in any country, it became apparent that all eligible studies were conducted in the U.S. As a result, we narrowed the scope of the review to fit with this U.S. focus, despite the fact that this was not our intention initially.

### 3.1. Study Characteristics

Table 1 describes the core information for each of the 15 included studies. All were conducted in the U.S., and all but one focused on treatment within VA medical centres and clinics [13]. The VA is America’s largest integrated health care system, providing care at 1243 facilities, and serving nine million ex-serving personnel per year [14]. Fourteen of the studies used electronic records to identify ex-serving personnel’s mental health service use [15,16,17,18,19,20,21,22,23,24,25,26,27,28]. Of these, eight accessed medical records retrospectively [16,22,23,24,25,26,27,28]. The final study in our review used a cross-sectional design, whereby participants completed self-reporting measures [13].

The average number of respondents per study was 31,782 (range = 97 [17]–309,050 [23]). All papers had mixed gender samples, although females typically made up a small percentage of participants (on average, 10.7% across the 15 studies; range = 3.6% [18]–23.0% [28]). This low proportion is reflective of the fact that enlisted women make up only 2.7% of the U.S. military’s front line units. Thirteen of the studies recruited ex-serving personnel who served in Iraq and/or Afghanistan only [13,15,16,17,18,19,20,21,22,23,24,25,26]. Clinical samples—that is, samples made up of participants with a clinical diagnosis/diagnoses, who visited mental health services—were recruited by 10 of the 15 included studies [15,16,17,19,20,22,23,24,27,28]. Of the remaining five studies, three included individuals attending an initial mental health assessment appointment [18,21,26]; one included individuals attending a compulsory post-deployment screening clinic [25]; and one included individuals from the community, identified through an ex-serving program roster [13].

### 3.2. Measures

Operationalisation of utilisation of mental health care services varied across papers. Eight studies solely focused on outpatient mental health care visits—including psychotherapy appointments, and psychiatric appointments—as their measure of mental health care utilisation [15,16,18,19,20,25,27,28]. Eleven papers implemented both categorical—dichotomous yes/no categorisation of utilisation of mental health care—and continuous—number of mental health care engagements—measures of mental health care utilisation within the given time frame [15,16,17,18,20,21,22,23,24,25,27]. Seven studies used a 12-month time frame over which mental health care utilisation was measured [15,18,19,20,23,24,25].

### 3.3. Rates of Mental Health Care Utilisation

To determine rates of mental health care utilisation, across these different measures and operationalisations, results were categorised into six sections (see Table 2). These six sections were made up of a two-by-three combination of the two measurement differences listed in the above paragraphs—(1) dichotomous versus continuous mental health care utilisation outcomes (two levels); and (2) outpatient mental health care visits versus inpatient psychiatric hospital stays versus medication use (three levels). The six categories were therefore as follows: (1) dichotomous, outpatient mental health care visits outcome; (2) dichotomous, inpatient psychiatric hospital stays outcome; (3) dichotomous, medication use outcome; (4) continuous, outpatient mental health care visits outcome; (5) continuous, inpatient psychiatric hospital stays outcome; and (6) continuous, medication use outcome. It is worth noting here that many studies had results relating to more than one of these six categories.

For category (1)—dichotomous, outpatient mental health care visits—the mean percentage prevalence rate of attending at least one outpatient mental health care visit was 63.2% across the 14 studies with relevant data, over an average time period of 10.4 months [13,15,16,17,18,19,20,21,22,23,24,25,27,28]. For category (2)—dichotomous, inpatient psychiatric hospital stays—the mean percentage prevalence rate of having at least one psychiatric inpatient episode was 12.5% across the two studies with relevant data, over an average time period of 12 months [23,24]. For category (3) —dichotomous, medication use—the mean prevalence rate of being prescribed medication was 43.5% across the four studies with relevant data, over an average time period of 4.5 months [13,17,19,20]. For category (4)—continuous, outpatient mental health care visits—the median number of outpatient mental health care visits attended was 7.6 across the 11 studies with relevant data, over an average time period of 13.1 months [15,16,17,18,20,22,23,24,25,26,27]. For category (5)—continuous, inpatient psychiatric hospital stays—the median number of inpatient psychiatric hospital stays was 0.1 across the three studies with relevant data, over an average time period of 16 months [23,24,26]. Finally, for category (6)—continuous, medication use—the number of days medication was taken was 247 for the one study with relevant data, over a time period of six months [22].

### 3.4. Associated Factors

Table 3, Table 4 and Table 5 show the mental health factors, the sociodemographic, military, and personality factors, and the treatment and functioning factors, respectively, associated with secondary mental health care utilisation.

As shown in Table 3, the most commonly reported statistically significant association, reported in six (out of eight) studies, was between PTSD severity and secondary mental health care utilisation [13,17,18,20,25,26]. Treatment utilisation occurred more frequently in those with more severe levels of PTSD. When PTSD was broken down by subscale, only the re-experiencing symptoms subscale was associated with utilisation of services. Three (out of three) studies reported a positive relationship between re-experiencing symptoms and treatment utilisation [15,18,22].

For the link between depression and treatment utilisation, mixed findings emerged—two studies reported a positive association [20,24], one a negative association [16], and three a non-significant association [18,22,26]. The mental health diagnosis of alcohol use disorder also had little support. It was positively associated with health care utilisation in one study [24], and non-significantly associated with health care utilisation in four studies [13,19,22,26]. Utilisation was significantly associated with comorbidity, with a positive relationship found in two (out of two) studies [19,24].

As shown in Table 4, the effect of age on secondary mental health care utilisation was unclear. Older age was positively associated with utilisation in three studies [16,27,28], and non-significantly associated with utilisation in three studies [13,19,21]. This was also the case for the sociodemographic variables of gender and ethnicity. Female sex and white ethnicity were positively associated with mental health care utilisation in two studies [15,20], and one [15] study, respectively. These same factors were negatively associated with mental health care utilisation in zero studies, and one study [23], respectively. However, seven studies testing associations for gender [13,16,19,24,27,28], and five testing associations for ethnicity [13,16,19,21,28] found no significant relationships between these constructs and treatment utilisation. The results for these factors were, therefore, inconclusive.

Alongside those already discussed, and as shown in Table 3, Table 4 and Table 5, a wide range of other mental health, sociodemographic, military, personality, treatment, and functioning factors were examined. None of these additional factors displayed consistent associations with secondary mental health care services.

## 4. Discussion

### 4.1. Key Findings

This narrative review found modest rates of secondary mental health care utilisation across the 15 included studies, indicative of moderate treatment-seeking in U.S. ex-military personnel. There was a large range in the frequency of mental health care utilisation across studies. These discrepancies are attributable to the large variation in how utilisation was defined and operationalised across the included studies, as well as differences in how the samples were recruited.

Two main factors were shown to be consistently associated with higher levels of secondary mental health care utilisation: PTSD (and re-experiencing symptoms in particular), and comorbidity. A number of other factors displayed mixed results in relation to service utilisation—perhaps most surprisingly, symptoms of depression and age.

### 4.2. Comparison to Previous Research

The mental health treatment utilisation rates found in this review are comparable to those reported in past studies of former military populations. For example, one paper reported that an average of 33% of U.S. ex-serving personnel previously deployed to Iraq or Afghanistan used inpatient and outpatient mental health services within the first year post-deployment [3]. Equivalent rates have been found when considering treatment-seeking within the U.S. civilian population [28,29,30,31]. We might expect lower rates of treatment utilisation in the military, given that certain socioeconomic groups at risk of not engaging with services are over-represented in this population (for example, younger men, and those from lower socioeconomic groups) [32]. However, as this is a group at high risk of mental health difficulties, it could be argued that they should be even more prepared to seek help than the general population [8]. 

As mentioned previously, it is worth noting that mental health care utilisation rates varied depending on the operationalisation of the term used. Most existing studies focusing on treatment utilisation in serving and ex-serving personnel have grouped both primary and secondary mental health care services together [6,7,8,22,33], which makes it difficult to assess rates of different types of help-seeking individually. An exception is U.S. research by Hoge and colleagues, which separates primary and secondary care, and reports that 28% of the included military sample engaged with secondary outpatient mental health clinics [5]. This number is substantially lower than the mean percentage prevalence rate of 63% found for those accessing secondary outpatient mental health visits in this review. Hoge and colleagues employed service members following deployment to Iraq or Afghanistan—some who had left service, and some who had not; whereas this review focused solely on ex-serving personnel [5]. We know that those no longer serving are more likely to be motivated to receive mental health care for depression, anxiety, and alcohol problems than those who are still serving [8,34], which may be increasing the rates of help-seeking in our review. Furthermore, Hoge and colleagues recruited a population-based military sample, who completed a routine post-deployment health assessment [5]; whereas the studies we included recruited predominantly from VA clinics, with a focus on individuals already engaging in treatment-seeking behaviour.

### 4.3. Associated Factors

The predominantly consistent associations found between PTSD and increased secondary mental health care utilisation are in line with past research investigating use of psychiatric services more generally [35]. This relationship is, arguably, unsurprising, given the link between PTSD and high rates of functional impairment, which then insist on a greater level of support than that provided in primary care settings. Recent studies examining predictors of mental health care use among ex-serving personnel have shown that greater PTSD symptom severity predicts greater service utilisation [36,37]. Former military members with a higher burden of mental health problems have a greater need for mental health care, and are, therefore, more likely to seek treatment [36]. However, two studies from the current review did not endorse the link between PTSD and mental health care utilisation [27,28]. Both of these papers included personnel who had last deployed to Iraq, Afghanistan, or another location, whereas the other 13 papers involved samples last deployed to Iraq and/or Afghanistan only. Perhaps the presence of PTSD and its accompanying symptoms vary depending on combat era, which in turn may elicit divergent responses in terms of secondary mental health care utilisation.

This review found that only the re-experiencing symptoms of PTSD were associated with higher levels of treatment-seeking, when measures were broken down further. Because of their direct association with trauma reminders from deployment, re-experiencing may facilitate both ex-serving individuals’ and health care providers’ recognition that the presenting distress is caused by PTSD, thus promoting more use of mental health services [13]. In contrast, other symptoms of PTSD (such as dysphoria or hyperarousal) might be attributed to psychosocial problems—for example, relationship difficulties; readjustment problems—for example, sleep habits learned on deployment; or medical problems—for example, musculoskeletal pain [13].

A positive significant association was found between secondary mental health care utilisation and comorbidity of mental health diagnoses. Indeed, this relationship is already well established within the literature [38,39]. Hines and colleagues have reported that meeting criteria for two or more, as opposed to none or one, mental health problems is associated with greater levels of psychological help-seeking in serving and ex-serving personnel [40]. Psychiatric comorbidity can be considered a proxy for patient distress [40], and, as discussed previously, ex-serving personnel are more likely to engage in mental health treatment as their distress, and their need for treatment, increases.

Unexpectedly, depression was not robustly linked with help-seeking. Earlier research by Possemato and colleagues has found a relationship between mental health conditions like depression and increased outpatient secondary mental health care utilisation in ex-serving personnel [39]. Studies included in this review focused only on those who were seeking help for a mental health problem. The ex-serving personnel included in the previous paper, on the other hand, were seeking help for either a mental or physical health difficulty [39]. Those with comorbid physical and mental health issues and poorer daily functioning have been shown to access mental health care treatment to a greater extent than those with mental health issues only and greater functioning [6,41]. 

The current review highlights mixed results for the association between secondary mental health care utilisation and age. This corresponds with past literature, showing that the relationship with age is unclear. For example, one study found that older age was associated with greater treatment engagement in Iraq and Afghanistan ex-serving personnel [41]. Another paper found that former military members under the age of 30 years are more likely to access mental health treatment than those above the age of 30 years when first screened positive for PTSD [42]. Still more have found a non-significant relationship between age and utilisation of mental health care services [33]. The inconsistent associations reported may be accounted for by a third mediation variable—for example, a number of papers have found that the influence of age can be accounted for by number of psychiatric disorders, number of trauma exposures, months since military separation, and rates of differing mental health diagnoses, including PTSD [19,43].

### 4.4. Strengths and Limitations

This comprehensive, multi-database narrative search and review into quantitatively measured secondary mental health care utilisation in U.S. ex-serving personnel used robust and well established methodology. However, considering the variability in the operationalisation of mental health care utilisation, our use of mean percentage prevalences and mean numbers to measure rates of engagement may not have been comparable across the included studies. Considering the 15 individual papers, all were conducted in the U.S. Furthermore, all contained a small minority of women. While statistical power to detect associations between gender and treatment-seeking may have, therefore, been limited in the papers included here, the low numbers reflect the low percentage of women who see active duty, and die in combat, in the U.S. military. Females are currently, however, the fastest-growing group of military veterans in the U.S., and future research should explore mental health care utilisation for this particular subpopulation in more detail. While a handful of the studies relied on self-reported measures of treatment utilisation, 11 made use of electronic health record registers. These registers are advantageous in providing rich material and numerous measurement points for large numbers of participants [44]. On the other hand, limitations of electronic systems include the large amounts of missing data often present [45,46], non-standardised clinical free-text notes [46,47], and a lack of information regarding undiagnosed mentally ill individuals [48]. 

### 4.5. Implications and Conclusions

The evidence presented here indicates that ex-serving personnel have modest levels of secondary mental health care treatment utilisation. Individuals engaged in outpatient care visits most often, most likely because these appointments are the most commonly offered source of support. Our results are comparable to those reported previously for both serving and civilian populations seeking secondary mental health care services. Utilisation of secondary mental health care was primarily related to more re-experiencing symptoms of PTSD, and more comorbid psychiatric conditions. Continued research is needed regarding the investigated factors that displayed mixed associations with secondary mental health care services, such as age, gender, and depression. Furthermore, we recommend the development of easily accessible interventions (for example, mobile health tools) that could contribute to facilitating substantially higher rates of help-seeking in ex-serving personnel. It is imperative that the overarching goal of research remains the successful reduction of distressing, clinically significant mental health problems. More specifically, it seems that the need to connect former military members suffering from non-PTSD-related diagnoses to mental health care treatment is substantial.

## Figures and Tables

**Table 1:** Core information for each included study.

Reference	*N*	Sample	Outcomes	Time Frame for Outcomes
**Samples deployed to Afghanistan/Iraq only**				
Blais et al. [15]	173	Ex-serving personnel with PTSD, enrolled in a VA post-deployment clinic for an initial evaluation.	Yes/No for attendance of two or more outpatient mental health care visits, for individual psychotherapy, group psychotherapy, and psychiatric visits. Number of outpatient mental health care visits, for individual psychotherapy, group psychotherapy, and psychiatric visits.	12 months, following initial assessment at clinic.
DeViva [16]	200	Ex-serving personnel with PTSD, referred to a PTSD specialist at one specific VA outpatient clinic.	Yes/No for attendance of outpatient mental health care visits, for psychotherapy sessions only. Number of outpatient mental health care visits, for psychotherapy sessions only. Number of days from referral to first scheduled outpatient visit.	No time frame specified.
DeViva et al. [17]	97	Ex-serving personnel with PTSD, referred to mental health services at one specific VA outpatient clinic.	Yes/No for attendance of outpatient mental health care visits, for psychotherapy sessions only. Yes/No for prescription of medication. Number of outpatient mental health care visits, for psychotherapy sessions only.	Six months, following referral to clinic.
Harpaz-Rotem et al. [18]	137	Ex-serving personnel, assessed at an initial scheduled screening appointment at one specific VA outpatient clinic.	Yes/No for attendance of outpatient mental health care visits. Number of outpatient mental health care visits.	12 months, following initial assessment at clinic.
Hearne [19]	429	Ex-serving personnel with any DSM axis 1 disorder, enrolled in a VA post-deployment clinic for an initial evaluation.	Yes/No for attendance of outpatient mental health care visits.	12 months, following initial assessment at clinic.
Hoerster et al. [20]	305	Ex-serving personnel with depression, PTSD, or alcohol misuse, enrolled in a VA post-deployment clinic for an initial evaluation.	Yes/No for attendance of nine or more outpatient mental health care visits, in line with minimally adequate treatment. Number of outpatient mental health care visits (including any in primary care VA settings).	12 months, following initial assessment at clinic.
Hudson et al. [21]	4782	Ex-serving personnel assessed at an initial scheduled appointment at a VA outpatient clinic.	Yes/No for attendance of outpatient mental health care visits, for psychotherapy sessions only. Yes/No for prescription of medication.	Three months, following initial assessment at clinic.
Kaier et al. [22]	124	Ex-serving personnel with PTSD or alcohol misuse, referred to study by primary care provider or VA case management team.	Yes/No for attendance of outpatient mental health care visits. Yes/No for prescription of medication. Number of outpatient mental health care visits. Number of days taking medication.	Six months, prior to referral to study.
Koo et al. [23]	309,050	Ex-serving personnel with PTSD, depression, anxiety, adjustment disorders, or alcohol or drug disorders, entered into VA care.	Yes/No for attendance of outpatient mental health care visits, and psychiatric inpatient stays. Number of outpatient mental health care visits, and psychiatric inpatient stays.	12 months, following initial assessment at clinic.
Maguen et al. [24]	159,705	Ex-serving personnel with PTSD, assessed at an initial scheduled appointment at a VA outpatient clinic.	Yes/No for attendance of outpatient mental health care visits, and psychiatric inpatient stays. Number of outpatient mental health care visits, and psychiatric inpatient stays.	12 months, following initial assessment at clinic.
McGinn et al. [25]	130	Ex-serving personnel in a committed relationship, enrolled in a VA post-deployment clinic for an initial evaluation.	Yes/No for attendance of mental health care visits, for outpatient psychiatric, and psychotherapy sessions (including any in primary care VA settings). Number of mental health care visits, for outpatient psychiatric, and psychotherapy sessions (including any in primary care VA settings).	12 months, following initial assessment at clinic.
Naragon-Gainey et al. [26]	618	Ex-serving personnel, assessed at an initial scheduled appointment at a VA outpatient clinic.	Number of mental health care visits, for mental health with primary health care visits, outpatient mental health care visits, and psychiatric inpatient stays.	24 months, following initial assessment at clinic.
Whealin et al. [13]	233	Ex-serving personnel from Hawaii, identified through an Iraq/Afghanistan era ex-serving program roster.	Yes/No for attendance of mental health care visits, for VA psychotherapy sessions, VA mental health care visits, and community mental health care visits. Yes/No for VA prescription of medication.	Three months, prior to entry into study.
**Samples deployed to Afghanistan/Iraq, as well as to other locations**				
Kehle-Forbes et al. [27]	427	Ex-serving personnel with PTSD, referred to, attended an initial assessment at, and put forward for further treatment at one specific VA outpatient clinic.	Yes/No for attendance of outpatient mental health care visits. Number of outpatient mental health care visits.	36 months, following referral to clinic.
Keller & Tuerk [28]	324	Ex-serving personnel with PTSD, assessed at an initial intake evaluation in one specific VA outpatient clinic, offered psychotherapy, and assigned a therapist to begin.	Yes/No for attendance of outpatient mental health care visits, for evidence-based PTSD treatment.	8 months, following assessment at clinic.

*N* = number of participants in the target study; PTSD = post-traumatic stress disorder; VA = U.S. Department of Veterans Affairs; DSM = Diagnostic and Statistical Manual of Mental Disorders, 5th edition.

**Table 2:** Rates of mental health care utilisation.

Classification	Outpatient MH Care Visits		Inpatient Psychiatric Hospital Stays		MH Medication Use	
	Reference	Rate (%) /Number	Reference	Rate (%) /Number	Reference	Rate (%) /Number
Dichotomous MH care outcome	**(1)**		**(2)**		**(3)**	
	Blais et al. [15]	90% *	Koo et al. [23]	13%	DeViva et al. [17]	32%
	DeViva [16]	62%	Maguen et al. [24]	12%	Hudson et al. [19]	77%
	DeViva et al. [17]	33%			Kaier et al. [20]	50%
	Harpaz-Rotem et al. [18]	73%			Whealin et al. [13]	15%
	Hearne [19]	53%				
	Hoerster et al. [20]	25% *				
	Hudson et al. [21]	52%				
	Kaier et al. [22]	68%				
	Kehle-Forbes et al. [27]	82%				
	Keller & Tuerk [28]	72%				
	Koo et al. [23]	93%				
	Maguen et al. [24]	96%				
	McGinn et al. [25]	50% *				
	Whealin et al. [13]	36%				
Overall mean rate		63.2%		12.5%		43.5%
Continuous MH care outcome	**(4)**		**(5)**		**(6)**	
	Blais et al. [15]	8.6	Koo et al. [23]	0	Kaier et al. [22]	247 *
	DeViva [16]	7.0	Maguen et al. [24]	0.1		
	DeViva et al. [17]	9.5	Naragon-Gainey et al. [26]	0.1		
	Harpaz-Rotem et al. [18]	14.7 *				
	Hoerster et al. [20]	7.5				
	Kaier et al. [22]	7.8				
	Kehle-Forbes et al. [27]	8.9				
	Koo et al. [23]	2.2				
	Maguen et al. [24]	6.4				
	McGinn et al. [25]	6.6				
	Naragon-Gainey et al. [26]	7.6				
Overall mean rate (SD)		7.9 (12.7)		0.1 (0.4)		247 (188)

MH = mental health; SD = standard deviation. The ‘Rate’ value represents: for category (1) the percentage prevalence rate of attending at least one outpatient mental health care visit, across the study’s sample and timeframe; for category (2) the percentage prevalence rate of having at least one psychiatric inpatient episode, across the study’s sample and timeframe; for category (3) the percentage prevalence rate of being prescribed medication, across the study’s sample and timeframe; for category (4) the average number of outpatient mental health care visits attended, across the study’s sample and timeframe; for category (5) the average number of inpatient psychiatric hospital stays, across the study’s sample and timeframe; and for category (6) the average number of days medication was taken, across the study’s sample and timeframe. * represents exceptions to these ‘Rate’ definitions: for category (1) Blais et al. [15] report the prevalence for those ex-serving personnel who attended two or more outpatient mental health care visits, Hoerster et al. [20] for those ex-serving personnel who attended nine or more, and McGinn et al. [24] for those ex-serving personnel who attended one or two; for category (4) Harpaz-Rotem et al. [18] report the average number of visits for those ex-serving personnel who attended at least one outpatient mental health care visit—whereas the other studies report the average number of visits for the whole sample of ex-serving personnel, including those who had accessed treatment, and those who had not; for category (6) Kaier et al. [22] report the number of days medication was taken, across the six month timeframe, and within this specific sample. Note: the 247 value is larger than 182.5—the number of days in six months—because a count of two is allocated if two different types of medication are taken on the same day.

**Table 3:** Mental health factors associated with mental health care utilisation for each included study, along with the direction of association.

Associated Factors	Significant Positive Associations	Non-Significant Associations	Significant Negative Associations
**Mental health factors**			
PTSD severity	DeViva et al. [17] ^1^ Harpaz-Rotem et al. [18] ^1^ Hoerster et al. [20] ^1^ McGinn et al. [25] ^4^ Naragon-Gainey et al. [26] ^4,5^ Whealin et al. [13] ^1^	Kehle-Forbes et al. [27] ^1^ Keller & Tuerk [28] ^1^	
Avoidance cluster		Harpaz-Rotem et al. [18] ^1^ Kaier et al. [22] ^4,6^	Blais et al. [15] ^4^
Dysphoria cluster		Blais et al. [15] ^4^	
Hyperarousal cluster		Blais et al. [15] ^4^ Harpaz-Rotem et al. [18] ^1^ Kaier et al. [22] ^4,6^	
Numbing cluster		Harpaz Rotem et al. [18] ^1^	
Re-experiencing cluster	Blais et al. [15] ^4^ Harpaz-Rotem et al. [18] ^1^ Kaier et al. [22] ^4,6^		
Depression	Hoerster et al. [20] ^1^ Maguen et al. [24] ^4,5^	Harpaz-Rotem et al. [18] ^1,4^ Kaier et al. [22] ^4,6^ Naragon-Gainey et al. [26] ^4,5^	DeViva [16] ^1^
Substance use disorder		DeViva [16] ^1^ Hearne [19] ^1^	
Alcohol use disorder	Maguen et al. [24] ^4,5^	Hearne [19] ^1^ Kaier et al. [22] ^4,6^ Naragon-Gainey et al. [26] ^4^ Whealin et al. [13] ^1^	
Traumatic brain injury		DeViva [16] ^1^ Whealin et al. [13] ^1^	
Comorbidity	Hearne [19] ^1^ Maguen et al. [24] ^4,5^		
Distress		Kaier et al. [22] ^4,6^	
Aggression		Naragon-Gainey et al. [26] ^4^	
Panic		Naragon-Gainey et al. [26] ^4^	

As described on page 6, the superscript numbers (running from 1 to 6) represent categories, incorporating two-by-three combinations of (1) dichotomous versus continuous mental health care utilisation outcomes (two levels); and (2) outpatient mental health care visits versus inpatient psychiatric hospital stays versus medication use (three levels). The six superscript categories are therefore as follows: (1) dichotomous, outpatient mental health care visits outcome; (2) dichotomous, inpatient psychiatric hospital stays outcome; (3) dichotomous, medication use outcome; (4) continuous, outpatient mental health care visits outcome; (5) continuous, inpatient psychiatric hospital stays outcome; and (6) continuous, medication use outcome. It is worth noting that many studies have results relating to more than one of these six categories. Various tests of association were used throughout the included studies.

**Table 4:** Sociodemographic, military, and personality factors associated with mental health care utilisation for each included study, along with the direction of association.

Associated Factors	Significant Positive Associations	Non-Significant Associations	Significant Negative Associations
**Sociodemographic factors**			
Female gender	Blais et al. [15] ^4^ Hoerster et al. [20] ^1^	DeViva [16] ^1^ Hearne [19] ^1^ Hudson et al. [21] ^1,3^ Kehle-Forbes et al. [27] ^1^ Keller & Tuerk [28] ^1^ Maguen et al. [24] ^5^ Whealin et al. [13] ^1^	
White ethnicity	Blais et al. [15] ^4^	DeViva [16] ^1^ Hearne [19] ^1^ Hudson et al. [21] ^1,3^ Keller & Tuerk [28] ^1^ Whealin et al. [13] ^1^	Koo et al. [23] ^1,2^
Older age	DeViva [16] ^1^ Kehle-Forbes et al. [27] ^1^ Keller & Tuerk [28] ^1^	Hearne [19] ^1^ Hudson et al. [21] ^1,3^ Whealin et al. [13] ^1^	
Married status	DeViva [16] ^1^	Whealin et al. [13] ^1^	
Employed status	DeViva [16] ^1^		
Non-student status		DeViva [16] ^1^	
Higher education level	Whealin et al. [13] ^1^	Harpaz-Rotem et al. [18] ^4^	
Urban living location		Hudson et al. [21] ^1^ Whealin et al. [13] ^1^	
Being a parent	Kaier et al. [22] ^4,6^		
Higher annual income	McGinn et al. [25] ^4^		
**Military factors**			
Combat exposure	Blais et al. [15] ^4^	Harpaz-Rotem et al. [18] ^1^	
Number of traumas experienced	Hearne [19] ^1^		
Military status (regular vs reserve)		Blais et al. [15] ^4^ DeViva [16] ^4^	
Branch of service		Hoerster et al. [20] ^1^	
Time since last deployment	Hearne [19] ^1^	DeViva [16] ^1^	
Number of deployments		DeViva [16] ^1^	
Service connection		Hudson et al. [21] ^1,3^	
Unit social support	Harpaz-Rotem et al. [18] ^1^		
Post-deployment social support		DeViva et al. [17] ^1^ Harpaz-Rotem et al. [18] ^1^	
Combat era		Keller & Tuerk [28] ^1^	
Fear of losing military-based vigilance		Harpaz-Rotem et al. [18] ^1^	
**Personality factors**			
Personality		DeViva et al. [16] ^1^	
Resilience		DeViva et al. [17] ^1^ Harpaz-Rotem et al. [18] ^1,4^	

As described on page 6, the superscript numbers (running from 1 to 6) represent categories, incorporating two-by-three combinations of (1) dichotomous versus continuous mental health care utilisation outcomes (two levels); and (2) outpatient mental health care visits versus inpatient psychiatric hospital stays versus medication use (three levels). The six superscript categories are therefore as follows: (1) dichotomous, outpatient mental health care visits outcome; (2) dichotomous, inpatient psychiatric hospital stays outcome; (3) dichotomous, medication use outcome; (4) continuous, outpatient mental health care visits outcome; (5) continuous, inpatient psychiatric hospital stays outcome; and (6) continuous, medication use outcome. It is worth noting that many studies have results relating to more than one of these six categories. Various tests of association were used throughout the included studies.

**Table 5:** Treatment and functioning factors associated with mental health care utilisation for each included study, along with the direction of association.

Associated Factors	Significant Positive Associations	Non-Significant Associations	Significant Negative Associations
**Treatment factors**			
Medication use		DeViva [16] ^1^	
Positive beliefs about mental health care	DeViva et al. [17] ^1^		
Barriers to mental health care		Hoerster et al. [20] ^1^	
Stigma regarding mental health care	DeViva et al. [17] ^1^	Hoerster et al. [20] ^1^ Whealin et al. [13] ^1^	
Type of treatment facility		Hudson et al. [21] ^1,3^	
Type of referral facility	Keller & Tuerk [28] ^1^	DeViva [18] ^1^	
Delivery of therapy		Kehle-Forbes et al. [27] ^1^	
Type of therapy		Kehle-Forbes et al. [27] ^1^	
Training level of treatment provider		Keller & Tuerk [28] ^1^	
Engagement in treatment outside VA		Hearne [19] ^1^	
Expressed interest in treatment outside VA		Hearne [19] ^1^	
Distance to the nearest VA clinic		Whealin et al. [13] ^1^	
**Functioning factors**			
Legal problems		DeViva [16] ^1^	
Social impairment		Kaier et al. [22] ^4,6^	
Relationship satisfaction			McGinn et al. [25] ^4^
Occupational impairment		Kaier et al. [22] ^4,6^	
Sleep quality		Harpaz-Rotem et al. [18] ^1^	
Pain		DeViva [16] ^1^ Naragon-Gainey et al. [26] ^4,5^	
Quality of life			Whealin et al. [13] ^1^
Life satisfaction		Harpaz-Rotem et al. [18] ^1^	

VA = U.S. Department of Veterans Affairs. As described on page 6, the superscript numbers (running from 1 to 6) represent categories, incorporating two-by-three combinations of (1) dichotomous versus continuous mental health care utilisation outcomes (two levels); and (2) outpatient mental health care visits versus inpatient psychiatric hospital stays versus medication use (three levels). The six superscript categories are therefore as follows: (1) dichotomous, outpatient mental health care visits outcome; (2) dichotomous, inpatient psychiatric hospital stays outcome; (3) dichotomous, medication use outcome; (4) continuous, outpatient mental health care visits outcome; (5) continuous, inpatient psychiatric hospital stays outcome; and (6) continuous, medication use outcome. It is worth noting that many studies have results relating to more than one of these six categories. Various tests of association were used throughout the included studies.
